# Uninvited guest, *Balantidium coli* in urine in a patient with bladder cancer: A case report and review of the literature

**DOI:** 10.1002/ccr3.7494

**Published:** 2023-07-17

**Authors:** Farnaz Farmani, Neda Soleimani, Mohammad Razeghi, Amir Zamani, Sahand Mohammadzadeh, Davoud Soleimani

**Affiliations:** ^1^ Department of Pathology, Shiraz Transplant Center, Abu Ali Sina Hospital Shiraz University of Medical Sciences Shiraz Iran; ^2^ Department of Pathology, Shiraz Medical School Shiraz University of Medical Sciences Shiraz Iran; ^3^ Center of Policy Planning Shiraz University of Medical Sciences Shiraz Iran

**Keywords:** balantidiasis, *Balantidium coli*, urinary balantidiasis, urine parasites

## Abstract

**Key Clinical Message:**

In contrast to intestinal balantidiasis, which is widespread throughout the world, urinary balantidiasis is uncommon. It often affects people with underlying diseases, and acute infections may be fatal. Even though urine is not typical for this parasite, specific morphologic characteristics can aid in accurate diagnosis.

**Abstract:**

*Balantidium coli* is a ciliated protozoan which can infect intestinal system. Urinary balantidiasis is an extremely rare infection that may cause serious issues in patients with underlying diseases. Herein, we present a case of urinary balantidiasis in a patient with bladder cancer.

## 1 BACKGROUND

*Balantidium coli* is a ciliated protozoan that can infect various mammals, including pigs and humans. Human infection (balantidiasis) is brought feco‐orally by consuming infective cysts with food and water. The trophozoites afterward mature and remain active in the intestinal lumen. Balantidiasis typically has no symptoms, but when it does, the manifestations include diarrhea, abdominal cramps, fever, nausea, and vomiting. More severe disease consequences and extraintestinal involvement often affect individuals with immune deficiency and underlying diseases.^1^, ^2^, ^3^, ^4^ Herein, we report a case of bladder cancer with *B. coli* in urine.

## 2 CASE PRESENTATION

A 71‐year‐old Iranian woman was transferred to our center with fever, chills, and flank pain for a week. One year earlier, she underwent transurethral resection of the bladder (TURB) for a bladder lesion. The pathologic report declared invasive high‐grade urothelial carcinoma, but she did not accept to do chemotherapy and surgery for cystectomy. After that, she was relatively well until 3 months ago when she presented with oliguria and abdominal pain. Sonography revealed severe bilateral hydronephrosis in addition to the advanced tumoral process in the pelvic cavity. Bilateral nephrostomy tubes were inserted, and she was discharged from the hospital. She had no history of abdominal cramp, diarrhea, nausea/vomiting, or contact with pigs. The patient had a history of hypertension (HTN) and Coronary Artery Disease (CAD) for 10 years, and she was taking carvedilol, aspirin, and losartan. She was a married housekeeper, post menopause, G5P5A0L5, without any history of smoking or alcohol consumption. The patient's psychological and family history was not significant.

On physical examination, blood pressure was 145/110 mmHg with a normal pulse rate of 88 beats per minute and a high temperature of 38.1°C. Chest examination was unremarkable. The abdomen was soft, but the right flank was tender, especially at the site of the nephrostomy tube. No organomegaly was identified. There was no significant problem on neurologic examination. With the possibility of nephrostomy tube infection, she was admitted for tube changing and further assessment.

The patient had normal blood urea nitrogen (BUN) and mildly elevated creatinine, 11^5^, ^6^, ^7^, ^8^, ^9^, ^10^, ^11^, ^12^, ^13^, ^14^, ^15^, ^16^, ^17^, ^18^, ^19^ and 1.5 (0.6–1.3) mg/dL, respectively. Serum fasting blood sugar (74 mg/dL) and complete blood count (CBC) [white blood cell (WBC): 7.4 × 10^3^/μL, hemoglobin: 10.1 g/dL, and platelet count: 286 × 10^3^/μL] were within normal ranges. Liver enzymes, including aspartate aminotransferase (AST), alanine aminotransferase (ALT), and alkaline phosphatase (ALP), were 16 (3–40), 8 (3–40), and 192 (80–306) IU/L, respectively. Serologic test for HIV antibody was negative.

**FIGURE 1:**
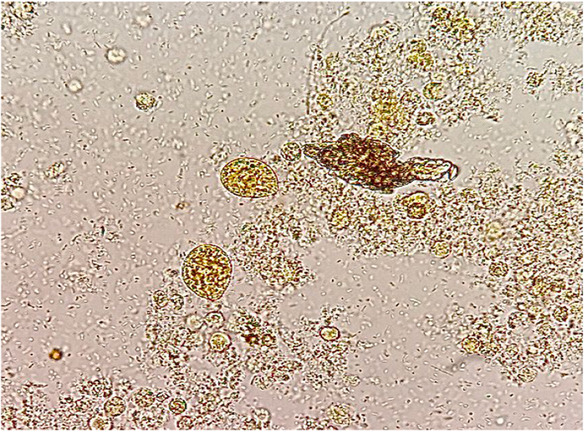
Urine sediment showing *Balantidium coli* trophozite (Lugol's iodine, 10× objective).

**FIGURE 2:**
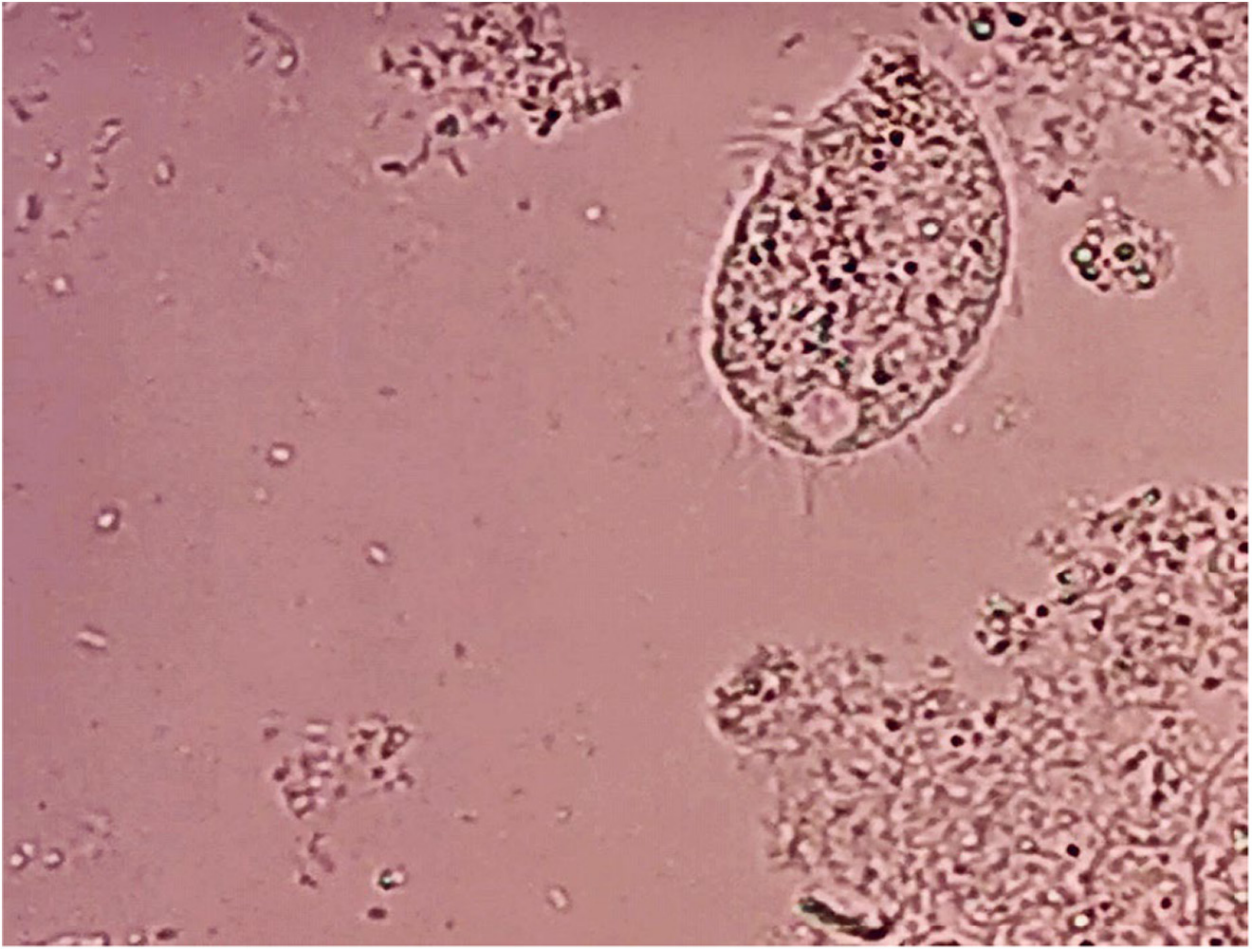
Urine sediment showing *Balantidium coli* trophozite with fain cilia (wet smear, 40× objective).

Microscopic urine analysis showed a moderate amount of red blood cells (RBCs) and WBC, as well as a moderate number of large ovoid‐shaped ciliated parasites (approximately 200 × 50 μm) swimming rapidly across the slide (Figure 1). The organism had a mouth in the tapering anterior end (cystosome) and a rounded posterior end (cytopyge). Several food vacuoles, macronucleus, and a few ingested RBSs were present within the cytoplasm. The body was covered with a pellicle with longitudinal striation and short, delicate cilia all around, of uniform length. The cilia lining the mouth part appeared longer than others (Figure 2). The morphology and the swimming pattern were characteristic of *B. coli*. A repeated urine sample showed similar parasites. Urine culture was negative for bacterial infection, but a stool sample was unavailable because of chronic constipation. Oral metronidazole was prescribed for 5 days (750 mg, three times daily), and subsequent urine samples during the 3‐month follow‐up visits showed clear urine with no RBC, WBC, and parasite, and she had no fever and flank tenderness. We obtained informed consent from the patient for publishing the case report and accompanying images. Also, our institutional approval was not required to publish the case details.

## 3 DISCUSSION

*Balantioides coli*, formerly known as *Balantidium coli*, is a ciliated protozoan that infects animals, including pigs, non‐human primates, and humans. Balantidiasis is the term for human infection. Having a global distribution, the prevalence of balantidiasis ranges from 0.02 to 1%^4^, ^20^

*Balantioides coli* is a common organism that is commonly found in the intestines of a broad range of insects, fish, amphibians, birds, and mammals. However, there are still cases of human balantidiasis in Muslims, which could indicate that another animal might serve as a reservoir host or that infection could spread due to direct transmission from human to human.^4^, ^5^, ^6^, ^7^, ^8^, ^20^

Consumed cysts release trophozoites that reside and replicate in the large bowel, primarily the cecum, though unusual extraintestinal colonization is also probable. It is believed that genitourinary site infections, such as uterine infection, vaginitis, and cystitis, are spread directly from the anal area or as a result of rectovaginal fistulas, which are developed due to *B. coli* infection. Additionally, it has also been reported to colonize the urinary tracts of dialysis patients.^1^, ^2^, ^3^, ^4^, ^9^, ^20^

Balantidiasis can affect several hosts, particularly those with chronic‐degenerative diseases or compromised immune systems. Immunodeficiency, alcoholism, and malnutrition are critical risk factors.^1^, ^10^, ^11^

Clinical signs can appear in one of three ways: Asymptomatic or occasional constipation and diarrhea, acute mucus‐bloody diarrhea, or chronic non‐bloody diarrhea alternating with constipation and non‐specific abdominal pains.^12^ Huge size, oval‐shaped, circumferential cilia and rapid spiral motion are the main morphological features of *B. coli* trophozoites, which facilitate diagnosis. In contrast to *Entamoeba histolytica* cysts, which are smaller (10–20 μm) and quadrinucleate, *B. coli* cysts are 40–60 μm in diameter and binucleate. The only other parasite with cilia and a similar appearance is *Paramecium*, which is nonpathogenic and can be found in contaminated water.^4^

The preferred treatment for *B. coli* is a combination of tetracycline and metronidazole.^4^, ^20^

Urinary balantidiasis is still a rare entity.^13^, ^14^ Genitourinary material for diagnosing evolutionary forms of *B. coli* should be collected with extreme care, using sterile collectors to avoid the contamination of urine samples with fecal matter, resulting in a false positive diagnosis.^15^

Until now, a few cases of urinary balantidiasis have been reported, and to the best of our knowledge, our case is the 14th case report of urinary balantidiasis.^16^ As shown in Table 1, most cases have been reported from India (seven cases), followed by Iran (three cases), Italy, Slovenia, Thailand, and Ethiopia (each reporting a single case). Cases with available data were analyzed. Most of the original countries were tropical, and most cases were female. The majority of patients were middle‐aged or older adults. Comorbidities frequently accompanied them, and two cases were pregnant at the time.

**TABLE 1:** Demographic and clinical data of reported cases of urinary balantidiasis.

Case no	Year	Country	Age(y/o) /Sex	Underlying disease	Presentation	Other significant findings	Outcome
1 Maleky^6^	1998	Iran	NA	NA	NA	NA	NA
2 Umesh^17^	2007	India	NA	NA	NA	NA	NA
3 Maino et al.^18^	2010	Italy	56/M	NHL	Acute kidney injury	–	Recovery
4 Bandyopadhyay et al.^19^	2013	India	72/F	–	Fever, dysuria, frequency, and pelvic pain	Intermittent diarrhea anemia	Recovery
5 Karuna et al.^21^	2014	India	68/M	DM/CKD	Fever, dysuria	–	Recovery
6 Khanduri et al.^22^	2014	India	55/F	–	Fever, malaise, anorexia, and oliguria		NA
7 Soleimanpour et al.^5^	2015	Iran	35/F	DM/hypothyroidism	Unrelated symptoms	Pregnancy	Recovery
8 Mane et al.^23^	2016	India	60/M	–	Fever, dysuria, frequency, abdominal pain, and oliguria	Positive urine culture for *E. coli*	Recovery
9 Gupta et al.^24^	2017	India	35/M	–	Fever, malaise, anorexia	–	Recovery
10 Gupta et al.^24^	2017	India	56/F	–	Anorexia, weakness and on and off diarrhea	–	Recovery
11 Tanja et al.^25^	2018	Slovenia	70/F	Psoriasis, HTN, dyslipidemia, DM, breast carcinoma, and Schwannoma of the stomach	Asymptomatic	–	Recovery
12 Martviset et al.^26^	2020	Thailand	48/F	SLE	Unrelated symptoms	–	Recovery
13 Almaw et al.^16^	2022	Ethiopia	24/F	–	Unrelated symptoms	Pregnancy	Recovery
Current case	2022	Iran	71/F	Bladder cancer, HTN, CAD	Fever, chills, and flank pain	–	Recovery

Abbreviations: CAD, coronary artery disease; CKD, chronic kidney disease; DM, diabetes mellitus; HTN, hypertension; NA, not available; NHL, non‐Hodgkin lymphoma; SLE, systemic lupus erythematous.

Table 2 compares all reported cases of urinary balantidiasis in terms of urinalysis data. Among available urinalysis data, macroscopic evaluation of most cases showed dark and turbid specimens with acidic pH and normal to low specific gravity, showing that this parasite could live and replicate in the normal acidic pH of urine. Moreover, some cases showed proteins, WBC, and RBC in urine, which could favor tissue irritation and inflammation of the urinary tract. Few patients had urinary casts and epithelial cells. Stool exams were negative in all cases, except for case 10, in which *B. coli* in urine was a false positive result of intestinal infection.

**TABLE 2:** Urinalysis data of reported cases of urinary balantidiasis.

Case no	Color	Appearance	Specific gravity	pH	Protein	Blood	Leukocyte esterase	RBC (/HPF)	WBC (/HPF)	Cast /LPF	Epithelial cell/HPF	Stool exam
1	NA	NA	NA	NA	NA	NA	NA	NA	NA	NA	NA	NA
2	NA	NA	NA	NA	NA	NA	NA	NA	NA	NA	NA	NA
3	NA	NA	1.008	5	−ve	−ve	−ve	−ve	−ve	Hyaline‐granular	+ve	−ve
4	NA	Turbid	NA	NA	NA	NA	NA	Many	5–6	NA	NA	−ve
5	NA	NA	NA	NA	NA	NA	NA	10‐12	15–20	Tubular	NA	−ve
6	Smoky	Turbid	NA	NA	NA	NA	NA	Few	2–4	NA	NA	−ve
7	Smoky	Turbid	NA	NA	Positive	Positive	−ve	50–60	20–25	Tubular	Few	−ve
8	NA	Turbid	1.008	5	−ve	−ve	−ve	Few	2–4	−ve	−ve	−ve
9	Smoky	Turbid	NA	NA	−ve	−ve	−ve	−ve	5–6	−ve	−ve	−ve
10	Yellow	NA	NA	Acidic	−ve	−ve	−ve	−ve	−ve	−ve	−ve	+ve
11	NA	NA	NA	NA	NA	NA	NA	NA	NA	NA	NA	−ve
12	Yellow	Turbid	1.04	6	4+	3+	2+	20–30	> 100	–	−ve	−ve
13	Dark	Turbid	1.01	6	1+	3+	2+	NA	Many	–	Many	−ve
Current case	Bloody	Turbid	1.004	6	1+	3+	−ve	Many	18‐20	–	4‐6	NA

Abbreviations: HPF, high power field; LPF, low power filed; NA, not available; RBC, red blood cell; WBC, white blood cell.

Our patient was an old woman with fever, chills, and flank pain. Routine urinalysis showed urinary balantidiasis, whereas there was no history of intestinal problems. Our patient's diagnosis of *B. coli* was based on clearly identified morphological features and motility. The organism, in this case, may have invaded the urinary tract through the colonic mucosa, directly through the anal area, or most probably from nephrostomy sites. Moreover, it has been estimated that old age and immune suppression are important factors favoring urinary balantidiasis.

This case is the third case report of urinary balantidiasis in Iran. Because of the Islamic prohibition on eating pork, piggeries in Iran, a Muslim country, were closed after the 1979 Islamic revolution, and pig breeding and pork consumption were banned. Therefore, the pig can be excluded as a source of infection in Iran. However, there are still reports of this infection, suggesting that a different animal, most probably wild boars, is a source of infection in Iran.^5^

## 4 CONCLUSION

In contrast to intestinal balantidiasis, which is widespread throughout the world, urinary balantidiasis is uncommon. It often affects middle‐aged or older people with underlying diseases (such as malignancy and immunodeficiency), and acute infections may be fatal. Even though urine is not typical for this parasite, specific morphologic characteristics can aid in a quick and accurate diagnosis of urinary balantidiasis.

## AUTHOR CONTRIBUTIONS

**Farnaz Farmani:** Conceptualization; data curation; formal analysis. **Neda Soleimani:** Conceptualization; data curation; investigation; methodology; supervision. **Mohammad Razeghi:** Investigation; methodology; resources; software. **Amir Zamani:** Validation; writing – original draft; writing – review and editing. **Sahand Mohammadzadeh:** Software; writing – original draft; writing – review and editing. **Davoud Soleimani:** Conceptualization; validation; writing – original draft; writing – review and editing.

## FUNDING INFORMATION

Not applicable since all authors are affiliated in Iran.

## CONFLICT OF INTEREST STATEMENT

The authors declare that they have no competing interests.

## ETHICS STATEMENT

Our institutional approval was not required to publish the case details.

## CONSENT

A written informed consent was obtained by the patient for publishing the case report and the publication of the accompanying images. A copy of the written consent is available for review by the Editor‐in‐Chief of this journal.

## Data Availability

All data generated or analyzed during this study are included in this published article.
